# A nested case–control study of predictors for tuberculosis recurrence in a large UK Centre

**DOI:** 10.1186/s12879-017-2933-4

**Published:** 2018-02-27

**Authors:** Andrew Rosser, Matthew Richardson, Martin J. Wiselka, Robert C. Free, Gerrit Woltmann, Galina V. Mukamolova, Manish Pareek

**Affiliations:** 1 Department of Infection, Immunity and Inflammation, University of Leicester, Leicester, LE1 7RH UK; 2 Department of Infection, Respiratory Biomedical Research Centre, Institute for Lung Health, Immunity and Inflammation, University of Leicester, Leicester, UK; 3 Department of Infection and Tropical Medicine, University Hospitals Leicester NHS Trust, Leicester, UK

**Keywords:** Tuberculosis recurrence, Tuberculosis reinfection, Tuberculosis relapse, *Mycobacterium tuberculosis*, Tuberculosis, TB

## Abstract

**Background:**

Tuberculosis (TB) recurrence represents a challenge to control programs. In low incidence countries, the prevailing risk factors leading to recurrence are poorly characterised.

**Methods:**

We conducted a nested case–control study using the Leicester TB service TBIT database. Cases were identified from database notifications between 1994 and 2014. Controls had one episode and were matched to cases on a ratio of two to one by the date of notification. Multiple imputation was used to account for missing data. Multivariate conditional logistic regression analysis was employed to identify clinical, sociodemographic and TB specific risk factors for recurrence.

**Results:**

From a cohort of 4628 patients, 82 TB recurrences occurred (1.8%). Nineteen of 82 patients had paired isolates with MIRU-VNTR strain type profiles available, of which 84% were relapses and 16% reinfections. On multivariate analysis, smoking (OR 3.8; *p* = 0.04), grade 3/4 adverse drug reactions (OR 5.6; *p* = 0.02), ethnicity ‘Indian subcontinent’ (OR 8.5; p = <0.01), ethnicity ‘other’ (OR 31.2; *p* = 0.01) and receipt of immunosuppressants (OR 6.8; p = <0.01) were independent predictors of TB recurrence.

**Conclusions:**

Within this UK setting, the rate of TB recurrence was low, predominantly due to relapse. The identification of an elevated recurrence risk amongst the ethnic group contributing most cases to the national TB burden presents an opportunity to improve individual and population health.

**Electronic supplementary material:**

The online version of this article (10.1186/s12879-017-2933-4) contains supplementary material, which is available to authorized users.

## Background

Tuberculosis (TB) treatment is often complicated by recurrence, defined as a further episode of TB occurring after a past episode had been declared cured [1]. The incidence of TB recurrence varies by location, with rates from 0.3 to 10.3 per 100 patient years (PYs) [2, 3]. Recurrence is caused by two distinct albeit clinically indistinguishable pathological processes, the endogenous reactivation of bacilli persisting after apparent cure is termed relapse, and exogenous infection by a new strain, reinfection [1]. Relapse is associated with chest x-ray (CXR) cavitation, drug resistance and poor adherence to treatment [4–6]. Human immunodeficiency virus (HIV) infection, migrant status, and a high local TB incidence predispose to reinfection [5, 7, 8]. Studies without access to molecular strain typing have elucidated many other risk factors including old age, failure to gain weight after treatment, and tobacco smoking [9–11]. Variations in risk factors between populations in part explains the preponderance of reinfection or relapse seen in different studies [12].

Minimizing recurrence should be a goal for every TB control program. TB recurrence has significant negative consequences for patients including retreatment expense, transmission, disease related morbidity and mortality [13–15]. When present, it indicates TB control and treatment activities need strengthening [16]. This can be best achieved by studying the local mechanisms of recurrence and identifying those groups at risk, so that preventative interventions are more precisely targeted [17].

At present, the understanding of TB recurrence in the UK is limited, being principally derived from the analysis of surveillance data by Crofts et al. covering the period 1998–2005 [18]. This approach is restricted in its ability to examine, in detail, the factors predisposing to recurrence within the population, to control for confounding, and to check the veracity of notification data to minimize information bias. In addition, the lack of access to molecular strain type profiles meant the magnitude of relapse and reinfection was unknown [18].

We postulated that by using a nested case–control study approach, considered to be an ideal design for studying TB recurrence [19], we could examine a wider range of clinical and demographic risk factors and identify those hitherto unrecognized within our local population, so better informing preventative strategies.

## Methods

### Study design and data source

All TB cases notified at the University Hospitals of Leicester NHS Trust (UHL), which serves the metropolitan area of Leicester (population 337,000 [20]; TB incidence 36.2 per 100,000 population per year [21]) and Leicestershire and Rutland (population, 705,000 [20]; TB incidence 3.4 per 100,000 population per year [21]) were prospectively recorded on a database (TBIT) used for TB case surveillance and management by the Leicester TB service. Locally, the investigation of patients with presumed TB follows guidelines produced by the National Institute for Health and Care Excellence (NICE) [22]. Those with presumptive pulmonary TB (PTB) initially undergo clinical assessment, sputum acid-fast bacillus (AFB) smear microscopy and culture and CXR and those with presumptive extrapulmonary TB (EPTB) may have site directed biopsy or needle aspiration. The ‘standard treatment’ regimen for patients with presumed or confirmed TB consists of 6 months of isoniazid and rifampicin with 8 weeks of pyrazinamide (with or without other drugs) added for the initial intensive phase of treatment. Where available drug susceptibility is used to guide TB treatment.

For the study, cases of TB recurrence were identified from patients notified twice or more to the database, controls were notified once. The new and first recurrence episode were used in the study. Subsequent recurrence episodes were not included. Patients were included if at the end of the first episode, they were designated treatment completed or cured [23]. TB nurse review confirmed completion of the allocated treatment course. Patients were excluded if during the first episode of TB they failed to complete treatment, transferred out-of-area, were lost to follow up, died or were classified as a treatment failure. Cases were matched to controls at a ratio of one to two by the date of notification with cases and controls separated by a maximum of 8 weeks.

### Study population and variables

All cases of TB notified to TBIT between 1st of January 1994 and 14th of December 2014 were included. Database entries were excluded where patients cultured non-tuberculous mycobacteria or were denotified due to subsequent reassignment to an alternative diagnosis other than TB.

The variables selected for analysis were significantly associated with recurrence in previous studies or considered biologically plausible. The data collected for variables such as alcohol or smoking status pertained to the prediagnostic and TB-treatment periods. Data were not available for the post-treatment period between the first and recurrent episode. Study data were extracted from the TBIT database, pseudonymised and recorded on an Access 2010 database (Microsoft, Redmond, Washington, USA). Reviews of paper and online records were performed to corroborate, supplement and fill in missing data. Further information pertaining to the study variables is presented in the Additional file 1.

### Definitions

TB definitions used in the study followed the World Health Organization definitions and reporting framework for TB [23]. A recurrent TB case was defined as ‘a patient who had been previously treated for TB, declared cured or treatment completed at the end of their most recent course of treatment and was diagnosed with a recurrent episode of TB (either a relapse or a new episode of TB caused by reinfection)’ [23]. This definition is independent of bacteriological confirmation. We defined ethnicity using the Office for National Statistics classification [24] (White, Indian subcontinent, Afro-Caribbean, Other Asia/Oriental, Other). As the proportion of ‘Other Asia/Oriental’ was small, this was subsumed into ‘Other’. Further details of definitions used are outlined in the Additional file 1.

### Molecular strain typing

Identification and typing of isolates was performed by the Health Protection Agency (now Public Health England) Midlands Regional Centre for Mycobacteriology. Restriction fragment length polymorphism (RFLP) typing of *Mycobacterium tuberculosis* (Mtb) strains was performed upon request until 2004, whereafter all isolates were analysed by mycobacterial interspersed repetitive units variable number tandem repeat (MIRU-VNTR) typing. Where available, MIRU-VNTR strain typing data were used to classify cases of recurrence [25]. If isolates from both episodes differed by two or more MIRU-VNTR loci, the case of recurrence was ascribed to reinfection and by one or less locus difference, relapse [8].

### Statistical analysis

Continuous and categorical variables were examined with the Wilcoxon rank-sum and Fisher’s exact test respectively to test for differences between cases and controls.

Multiple imputation by chained equations was performed. Variables imputed included weight, habitual alcohol use, habitual tobacco smoking, UK-birth status, blood haemoglobin concentration, white cell count, serum creatinine, acid fast bacillus smear status, TB culture status and phenotypic antimicrobial susceptibility (sensitive/resistant). The median percentage of missing data for imputed variables was 9.6% (IQR 4.9–12.2%; range 1.6–39.4%). All variables used in the analysis model plus the dependent variable were included in the imputation model. Vitamin D and Bacillus Calmette–Guérin variables were missing approximately 50% of data causing model instability and were excluded. Duration of treatment was also excluded for model instability. A burn-in period of 100 was used between imputations. 60 imputation sets were created for conditional logistic regression analysis. No interactions were specified in the imputation model. Post imputation analysis showed good evidence of convergence.

A multivariate conditional logistic regression model was fitted with variables as potential predictors using recurrence of TB as the main outcome variable (see Table 3). Before analysis, the presence of multiple collinearity was considered. The variables ‘standard treatment’ and ‘treatment interruption’ both exhibited collinearity with ‘adverse drug reaction’. The variable ‘other comorbidities’ also exhibited collinearity with ‘creatinine’. Those variables exhibiting collinearity were excluded. Model specification errors were screened for using link test. A *p*-value of <0.05 was considered significant. Statistical analyses were performed using Stata 13.1 statistics package (Statacorp, Texas, USA). Case–control matching was performed by SPSS version 22 (IBM, Redmond, USA).

The recurrence incidence rate after the first TB episode was calculated from the end of treatment. The estimated reinfection rate was calculated supposing those patients without typing data had recurrent disease due to reinfection in the same proportions as those for whom typing data was available. The same approach was taken for the estimated relapse rate.

## Results

### Description of study cohort

**Fig. 1:**
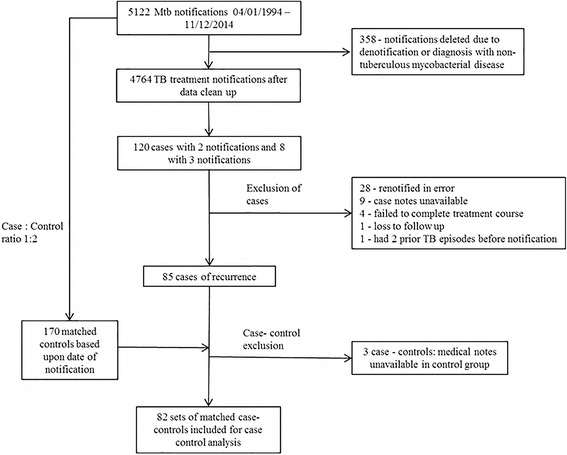
Flow diagram of study cases and controls included in the data analysis

The study flow chart describing patient recruitment is outlined in Fig. 1 and the demographic and clinical details of cases and controls in Table 1. Compared to controls, cases were more likely to habitually consume alcohol, habitually smoke tobacco, have AFB smear positive clinical samples and to receive immunosuppressant drugs (see Additional file 1: Table S1 for the immunosuppressive medication received and indications).

**Table 1:** Cohort demographics

	Case (*n* = 82) N (%)	Control (*n* = 164) N (%)	*P* value
Age (year, +/−SD)	40.2 +/−17.6	37.4 +/−17.6	0.23
Weight (kg)^a^	56 (45.5–68)^**^	59 (48–68)^**^	0.26
Gender			
Male	42 (51.2)	84 (51.2)	–
Female	40 (48.8)	80 (48.8)	1.00
Ethnicity			
White	10 (12.2)	20 (12.2)	–
Afro-Caribbean	7 (8.5)	17 (10.4)	0.78
Indian subcontinent	61 (74.4)	125 (76.2)	1.0
Other	4 (4.9)	2 (1.2)	0.18
Permanent residence	81 (98.8)	163 (99.4)	1.00
UK Born^b^	25 (30.5)	37 (22.5)	0.16
Habitual alcohol consumption^c^	17 (23.6)	20 (12.4)	0.03
Habitual tobacco smoking^d^	20 (26.3)	19 (11.7)	0.01
BCG vaccinated^e^	31 (79.5)	73 (81.1)	0.81
Comorbidities			
Diabetes mellitus	9 (11.0)	15 (9.2)	0.65
Solid organ transplant±	0	1 (0.6)	1.00
Cancer	0	3 (1.8)	0.55
End stage renal failure	1 (1.2)	3 (1.8)	1.00
Rheumatological conditions^**¶**^	3 (3.7)	1 (0.6)	0.11
HIV-1 seropositivity	5 (6.1)	6 (3.7)	0.51
Vitamin D deficiency^f^	24 (64.8)	52 (69.3)	0.67
Laboratory			
Haemoglobin^g^ (g/dl +/−SD)	11.7+/−1.8	12.2+/−1.9	0.11
White cell count^h^ (×10^9^ cells/L)	7.5 (6.0–9.6)^**^	7.7 (5.9–9.5)^**^	0.91
Creatinine^i^ (μmol/l)	70.0 (61.5–79.0)^**^	71.0 (61.0–83.0)^**^	0.46
Primary disease site			
PTB	45 (54.8)	68 (41.4)	–
PTB + EPTB	10 (12.2)	31 (19.0)	0.09
EPTB	27 (33.0)	65 (39.6)	0.14
Pulmonary cavitation	19 (23.2)	23 (14.0)	0.11
Microbiology			
AFB smear positive^j^	30 (41.7)	42 (27.7)	0.01
Mtb culture positive^k^	56 (76.7)	93 (66.9)	0.16
Drug resistance^l^	7 (12.5)	6 (6.5)	0.24
Treatment			
Standard regimen	68 (82.9)	145 (88.4)	0.24
ADR			
No ADR	64 (78.0)	143 (87.2)	–
Grade 1/2	4 (4.9)	7 (4.3)	0.74
Grade 3/4	14 (17.1)	14 (8.5)	0.06
Treatment interruption (days)	0 (0–0)^**^	0 (0–0)^**^	0.96
Immunosuppressing drugs	17 (20.7)	16 (9.8)	0.03
Duration (days)	168 (168–273)^**^	168 (168–274)^**^	0.50

The missing data for case and controls is:

^a^14&16; ^b^2&2; ^c^10&2; ^d^6&2; ^e^4374; ^f^45&89; ^g^8&15; ^h^8&16; ^i^6&17; ^j^10&19; ^k^9&25; ^l^26&71

*SD* standard deviation, *HIV* human immunodeficiency virus, *PTB* pulmonary tuberculosis, *EPTB* extrapulmonary tuberculosis, *ADR* adverse drug reaction, *AFB* acid fast bacillus, Mtb *Mycobacterium tuberculosis, BCG* bacillus Calmette–Guérin

**±**Renal transplant' ±Renal transplant' to its own line

^**¶**^Psoriasis and rheumatoid arthritis

^**^median value and interquartile range

The pulmonary tract was the major site of disease for those with and without recurrence. Lymph node disease was the predominant EPTB type (see Additional file 1: Table S2 for the anatomical location of TB disease in cases and controls). 4.8% of patients with recurrence died.

### Incidence and duration of recurrence

**Fig. 2:**
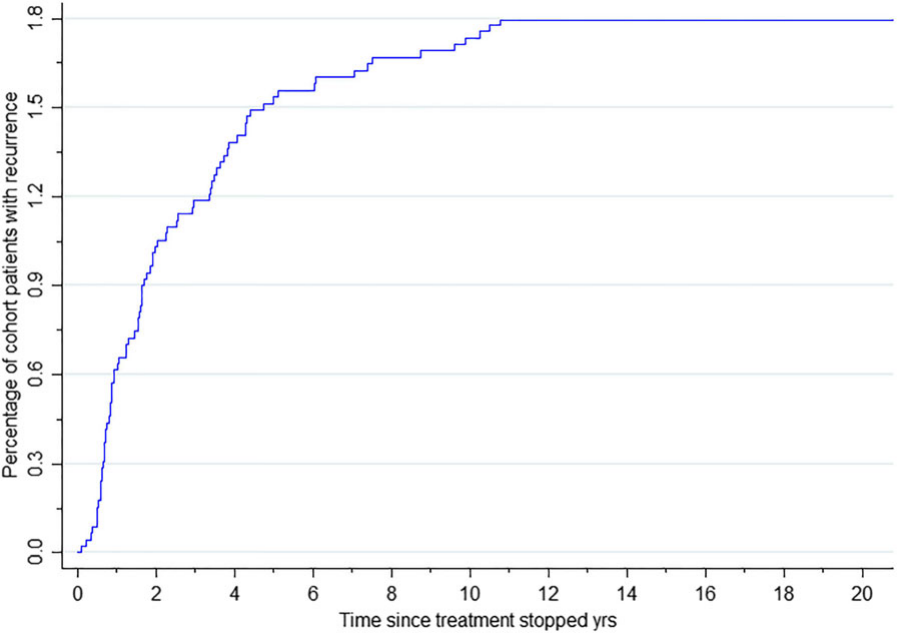
Kaplan-Meier curve showing cumulative percentage recurrence in 4628 notified tuberculosis patients

Figure 2 demonstrates that 85 patients out of a total study cohort of 4764 (1.8%) experienced recurrence. The median observational period was 7.8 years (IQR 3.6–11.6 years; range 0.7–20.8 years). The median time to recurrence after treatment completion was 1.7 years (IQR 0.8–3.8 years; range: 0.1–10.8 years). 57.3% (95%CI 46.5–67.5%) of recurrence cases were diagnosed within 2 years and 76.8% (95%CI 66.6–84.6%) within 4 years after treatment completion. The overall recurrence incidence rate was 1.9 (95%CI 1.5–2.3) per 1000 patient years of follow up (PYs) with the incidence highest within 6–12 months post TB treatment (9.9 (95%CI 6.4–14.8) per 1000PYs) and subsequently decreasing during the 1–2 year (4.5 (95%CI 2.8–6.9) per 1000PYs) and 2–4 year periods (2.1 (95%CI 1.2–3.3) per 1000PYs) post TB treatment.

### Reinfection versus relapse

Eighty-five cases of recurrence were identified of which three were excluded (see Fig. 1). Of the remaining 82 cases, paired MIRU-VNTR strain-type profiles were available for 19 isolates. No RFLP data were available. There were three (16%; 95%CI 3.4–39.6%) cases of reinfection and 16 (84%; 95%CI 60.4–96.6%) of relapse (see Additional file 1: Table S3 for strain typing data). No significant differences in clinical or demographic characteristics between relapse and reinfection cases were found (see Additional file 1: Table S4). The median time to recurrence was 0.8 years (IQR 0.5–0.9; range 0.2–1.1 years) for confirmed reinfection cases and 1.4 years (IQR 0.7–2.4; range 0.1–5.1 years) for confirmed relapses (*p* = 0.27). The estimated reinfection incidence rate was 0.3 (95% CI 0.2–0.5) per 1000PYs. The estimated relapse rate was 1.6 (95% CI 1.2–2.0) per 1000PYs.

### Drug resistance

Table 2 presents the sensitivity profiles of 29 recurrence cases where paired cultures were available. Cases of reinfection demonstrated no change in sensitivity profile between the primary and recurrence episode. Of the 16 cases of relapse, 13 (81%) displayed no change in sensitivity profile and 3 (19%) decreased susceptibility to one additional drug with one isolate becoming a multidrug resistant strain.

**Table 2:** The change in sensitivity profile in recurrence patients with paired isolates

Recurrence classification	Number of cases (29)	Primary episode sensitivity profile	Recurrence episode sensitivity profile
Recurrence	10	Sensitive	Sensitive
Reinfection	2	Sensitive	Sensitive
	1	H resistant	H resistant
Relapse	11	Sensitive	Sensitive
	1	Sensitive	R resistant
	1	Q & H resistant	H resistant (Q not tested)
	1	H resistant	RH resistant
	1	RHZQ resistant	RHZEQ resistant
	1	RHZE resistant	RHZE resistant

*R* rifampicin, *H* isoniazid, *Z* pyrazinamide, *E* ethambutol, *Q* Quinolone

Recurrence: two episodes of tuberculosis characterized by paired cultures positive for Mtb without MIRU-VNTR strain typing performed; Relapse: ≤ 1 MIRU-VNTR locus difference between isolates; Reinfection: ≥2 MIRU-VNTR loci differences

### Factors associated with recurrence on conditional logistic regression analysis

Univariate and multivariate associations of factors linked to TB recurrence are displayed in Table 3. Univariate conditional logistic regression analysis showed habitual alcohol and tobacco consumption, AFB smear positivity, receiving immunosuppressants and suffering a grade 3/4 adverse drug reaction (ADR) significantly increased the risk of TB recurrence. Controlling for confounding with multivariate analysis revealed that ethnicity ‘Indian subcontinent’ (odds ratio (OR) 8.5, 95% confidence interval (95% CI) 1.8–39.3, p = <0.01), ethnicity ‘Other’ (OR 31.2, 95% CI 2.1–471 *p* = 0.01), habitual tobacco smoking (OR 3.8, 95%CI 1.1–13.1, *p* = 0.04), a grade 3/4 ADR (OR 5.6, 95%CI 1.3–24.8, *p* = 0.02) and receiving immunosuppressants (OR 6.8, 95% CI 1.9–24.4; p = <0.01) significantly increase the risk of TB recurrence.

**Table 3:** Factors associated with tuberculosis recurrence on conditional logistic regression

	Unadjusted Odds ratio (95% CI)	*P* value	Adjusted odds ratio (95% CI)	*P* value
Age	1.0 (1.0–1.0)	0.26	1.0(1.0–1.1)	0.09
Weight	1.0 (1.0–1.0)	0.29	1.0 (1.0–1.0)	0.46
Gender				
Male	Reference		Reference	
Female	1.0 (0.6–1.7)	1.00	1.1(0.5–2.7)	0.85
Ethnicity				
White	Reference		Reference	
Afro-Caribbean	0.8 (0.3–2.7)	0.75	5.4 (0.7–43.4)	0.13
Indian subcontinent	1.0 (0.4–2.2)	0.96	8.5(1.8–39.3)	<0.01
Other	3.9 (0.6–24.8)	0.15	31.2(2.1–471.0)	0.01
UK Born	1.5 (0.8–2.8)	0.17	2.8(0.9–8.8)	0.07
Permanent residence	0.5 (0.3–8.0)	0.62	0.2(0.0–4.0)	0.26
Habitual alcohol consumption	2.2 (1.1–4.8)	0.03	1.8(0.6–5.7)	0.30
Habitual tobacco smoking	2.8 (1.4–5.6)	<0.01	3.8(1.1–13.1)	0.04
Co-morbidities				
Diabetes mellitus	1.2 (0.5–2.7)	0.67	0.9 (0.2–2.8)	0.86
HIV-1 seropositivity	1.7 (0.5–5.5)	0.40	4.1(0.7–23.1)	0.12
Other comorbidities±	1.0 (0.3–3.5)	1.00	a	
Laboratory				
Haemoglobin (g/dl)	0.9 (0.8–1.0)	0.09	0.9 (0.7–1.2)	0.54
White cell count (×10^9^ cell/l)	1.0(1.0–1.1)	0.44	1.0 (0.8–1.1)	0.36
Creatinine (μmol/l)	1.0 (1.0–1.0)	0.86	1.0(1.0–1.0)	0.41
Primary disease site				
PTB	Reference		Reference	
PTB + EPTB	0.5 (0.2–1.1)	0.09	0.7 (0.2–2.1)	0.49
EPTB	0.6 (0.4–1.1)	0.12	1.4 (0.6–3.7)	0.49
Pulmonary cavitation	1.8 (0.9–3.6)	0.08	1.3 (0.5–3.9)	0.61
Microbiology				
AFB positive	2.3 (1.2–4.1)	<0.01	1.7 (0.6–4.3)	0.30
Culture positive	1.6 (0.8–2.9)	0.16	1.3 (0.5–3.2)	0.54
Drug resistant	1.5 (0.5–4.1)	0.49	2.6 (0.5–13.8)	0.28
Treatment				
Standard regimen	0.7(0.3–1.4)	0.26	a	
ADR				
No ADR	Reference		Reference	
Grade 1/2	1.2 (0.4–4.1)	0.78	1.3 (0.3–6.1)	0.78
Grade 3/4	2.3 (1.0–5.3)	<0.05	5.6 (1.3–24.8)	**0.02**
Treatment interruption	1.1 (0.4–2.6)	0.88	a	
Immunosuppressing drugs	3.0 (1.2–7.1)	0.01	6.8 (1.9–24.4)	<0.01

±Active cancer, end stage renal failure, solid organ transplant & rheumatology conditions

*HIV* human immunodeficiency virus, *PTB* pulmonary tuberculosis, *EPTB* extrapulmonary tuberculosis, *ADR* adverse drug reaction, *AFB* acid fast bacillus

^a^ not included in the analysis due to multiple collinearity

## Discussion

We conducted a nested case control study within an ethnically diverse UK setting to uncover factors associated with TB recurrence. We found in patients who completed standard treatment for their first TB episode, that ethnicity (Indian subcontinent and other ethnic groups), habitual tobacco smoking, receipt of immunosuppressant medication and suffering a grade 3/4 ADR independently increased the risk of subsequent TB recurrence. Recurrence itself was an infrequent occurrence affecting 1.8% of the study cohort with a peak incidence at 6 to 12 months after treatment completion. Relapse (endogenous reactivation) was the predominant mechanism of TB recurrence affecting 84% of patients for whom paired molecular strain-type profiles were available.

The overall TB recurrence incidence in Leicester of 1.9 per 1000PYs was low, comparable to the rate of 4.1 per 1000PYs for England and Wales [18] and to other low incidence countries such as Australia (0.8 per 1000PYs [26]) and Spain (5.3 per 1000PYs [3]). We observed that 77% of recurrences occurred within 4 years after treatment completion. This contrasts with the findings of Nunn et al. who found a 91% recurrence rate at 12 months [27] and may be explained by our longer duration of follow up and the treatment of patients outside a clinical trial setting delaying diagnosis.

We established that Indian subcontinent and other ethnicity were significantly associated with TB recurrence and Afro-Caribbean ethnicity demonstrated a positive although non-significant trend. Prior studies demonstrated significant associations between non-white ethnicity and both TB disease phenotype [28] and severity [29]. In the present study, we were unable to determine whether ethnicity (host genotype) itself mediates an individual’s risk of recurrence or functions as a proxy for other risk factors(s). Certain ethnic groups have a heightened exposure to TB, for example from travel to high incidence areas to visit family and friends or due to poor housing within migrant communities. However, this remains theoretical in the absence of robust data. Additionally, there may be differences between ethnic groups such as vitamin D status, ability to access health care or adherence to treatment which were unaccounted for in our study and could mediate the association we demonstrated. The strong association between TB recurrence and Indian subcontinent ethnicity, a group that are the most important in terms of TB cases nationally [30] and contribute more than 25% of cases worldwide [31], may have significant implications for individual patient management and public health. Conceivably interventions targeting this large at-risk group could significantly prevent future TB episodes. Future work should focus on the factors underpinning the association between ethnicity and TB recurrence.

In this study, experiencing a grade 3/4 adverse drug reaction to TB treatment elevated a patient’s future risk of TB recurrence. ADRs are considered a major cause of poor adherence to TB treatment [32], which in turn is associated with an increased risk of TB recurrence [14]. Unfortunately, data detailing patient adherence were not routinely recorded and were thus not controlled for in multivariate analysis. Although not directly examining TB recurrence, a study conducted in an MDR-TB cohort showed that ADRs did not impact treatment outcomes in those patients adherent to treatment [33]. Adherence may therefore mediate the observed association between ADRs and TB recurrence although further research is required to explore this.

We found habitual tobacco smoking to independently predict TB recurrence. This reflects findings by others [34] and identifies a problem area within our cohort. Smoking increases baseline disease severity and reduces treatment response through inhibiting macrophage immune function [34, 35] possibly increasing recurrence risk through immunoparesis. Smoking cessation strategies should be pursued in all TB patients to minimize recurrence as well as for the other accepted health benefits.

Immunosuppression of any kind predisposes to TB [36]. We found for the first time that receiving immunosuppressants anytime from symptom onset until completion of TB treatment in the first TB episode predisposed to recurrence. Most patients received immunosuppressants for appropriate indications including treatment of co-morbid conditions and the complications of TB, however a minority were for incorrect diagnoses or complications of TB where no significant evidence of benefit existed. Consequentially, we advocate the judicious use of immunosuppressants in patients with suspected or confirmed TB with careful consideration of the risk: benefit ratio.

To date no study has detailed the proportion of recurrence due to relapse and reinfection in the UK. We found relapse to be the predominant mechanism accounting for 84% of recurrences although a larger dataset is required to accurately quantify the relative proportion of relapse and reinfection. The number of typed isolates was insufficient to permit examination of whether risk factors were specific to reinfection, relapse or both.

Our work had several limitations. First, the information on several confounding factors (e.g. smoking) was based upon self-reported data and may not represent actual exposure status, although this is likely to have affected cases and controls equally. Likewise, adherence was self-reported and poorly recorded in the medical notes so could not be accounted for in multiple imputation and multivariate analysis. Additionally, several potential confounders (e.g. vitamin D status), with high fractions of missing information or exhibiting collinearity, lead to imputation model instability and were excluded. It is possible that the omission of potential confounding variables may have misspecified the imputation model and biased subsequent analysis. Second, misclassification bias may have been introduced into the study. Patients moving out of the hospital catchment area or renotification failures may have underestimated the number of recurrences and the inclusion of culture negative cases may lead to overestimates. Cases of reinfection and relapse may have been misclassified by the use of low resolution MIRU-VNTR strain-type [37] and future studies of recurrence in the UK should utilise higher resolution whole genome sequencing. Finally, as paired molecular strain-type information was available for only 19 (23%) patients, the 95% confidence intervals for the relative proportion of recurrence due to relapse and reinfection were broad.

## Conclusion

We found TB recurrence to be uncommon, although for a proportion of patients affected, the consequences were severe. Several risk factors we identified may be addressed through established clinical strategies although research is required to show risk factor modification reduces recurrence and how it is best implemented. The attendant risk amongst ‘Indian subcontinent’ and ‘other’ ethnic groups, who together comprise the largest burden of TB nationally, has substantial implications for individual patient management as well as for public health. Future work should incorporate host genetic, socioeconomic and environmental factors to unpick this association to inform future preventative strategies.

## Additional file

Additional file 1: Summary of definitions used in the study. Tables detailing: immunosuppressive medication received by patients; site of tuberculosis disease for cases and controls; MIRU VNTR strain type data for paired isolates; demographic data for reinfection and relapse cases. (DOCX 107 kb)

## Abbreviations

ADR: Adverse drug reactions
AFB: Acid fast bacillus
EPTB: Extrapulmonary tuberculosis
HIV: Human immunodeficiency virus
IQR: Interquartile range
MIRU-VNTR: Mycobacterial interspersed repetitive units variable number tandem repeat
OR: Odds ratio
PTB: Pulmonary tuberculosis
PY: Patient years
TB: tuberculosis
