# Supplementary material for: A nested case–control study of predictors for tuberculosis recurrence in a large UK Centre
Source: BMC Infect Dis. 2018 Feb 27;18:94. doi: 10.1186/s12879-017-2933-4 (PMC5830048; doi:10.1186/s12879-017-2933-4)
Supplement: Supplementary file 1 — Summary of definitions used in the study. Tables detailing: immunosuppressive medication received by patients; site of tuberculosis disease for cases and controls; MIRU VNTR strain type data for paired isolates; demographic data for reinfection and relapse cases. (DOCX 107 kb) [file 12879_2017_2933_MOESM1_ESM.docx]

**A nested case-control study of predictors for tuberculosis recurrence in a large UK centre**

Andrew Rosser^1,3^; Matthew Richardson^2^; Martin Wiselka^3^; Robert C. Free^2^, Gerrit Woltmann^2^; Galina Mukamolova^1^; Manish Pareek^1,3^

^1^Department of Infection, Immunity and Inflammation, University of Leicester, Leicester, UK

^2^Respiratory Biomedical Research Centre, Institute for Lung Health, Department of Infection, Immunity and Inflammation, University of Leicester, Leicester, UK

^3^Department of Infection and Tropical Medicine, University Hospitals Leicester NHS Trust, Leicester, UK

**Corresponding Author** Dr Andrew Rosser

Department of Infection and Tropical Medicine, Leicester Royal Infirmary, Infirmary Square, Leicester, LE1 5WW. UK.

Email: [andrew.rosser@gmail.com](mailto:andrew.rosser@gmail.com)

**Supplementary methods**

**Definitions**

- *A case of tuberculosis*: Where a biological specimen is positive for Mtb by smear microscopy, culture or an approved rapid diagnostic test or a case which has been diagnosed as active Mtb by a clinician and a full course of treatment is planned to be administered [[1](#_ENREF_1)].
- *Pulmonary tuberculosis (PTB):* Disease involving the lung parenchyma or tracheobronchial tree [[1](#_ENREF_1)].
- *Extrapulmonary tuberculosis (EPTB):* Any case not involving the lungs such as the brain, intrathoracic lymph nodes or pleura [[1](#_ENREF_1)].
- *Recurrence:* A patient who has been treated for TB disease declared cured or treatment completed at the end of treatment and is now diagnosed with a recurrent episode of Mtb disease whether that be relapse or reinfection [[1](#_ENREF_1)].
- *Reinfection:* A second episode of tuberculosis resulting from exogenous infection with an Mtb strain differing from that responsible for the first episode by ≥ 2 MIRU-VNTR loci [[2](#_ENREF_2), [3](#_ENREF_3)].
- *Relapse:* A second episode of tuberculosis resulting from endogenous reactivation of the Mtb strain responsible for the first episode as shown by ≤ 1 MIRU-VNTR locus difference [[2](#_ENREF_2), [3](#_ENREF_3)].
- *Standard treatment regimen:* the use of at least six months of isoniazid and rifampicin with 8 weeks of pyrazinamide (with or without other drugs).

Demographics:

- Ethnicity: classified by the Office for National Statistics classification (White, Indian Subcontinent, Afro-Caribbean, Oriental / Other Asia, Other) [[4](#_ENREF_4)].
- *Housed:* Resident at a fixed address.
- *Smoker:* the regular smoking of tobacco at the time of diagnosis.
- *Alcohol user:* habitual alcohol consumption at the time of diagnosis.

Co-morbidities:

- *Diabetes mellitus:* the documented use of diabetic medication [[5](#_ENREF_5)], a fasting blood sugar ≥7.0mmol/L, a 2 hour blood glucose of ≥11.1mmol/L after an oral glucose tolerance test, an HB_A1C_ of ≥6.5% or symptoms of hyperglycaemia with a random blood sugar ≥11.1mmol/L [[6](#_ENREF_6)].
- *End stage renal failure:* an estimated glomerular filtration rate (eGFR) of <15ml/min/1.73m^2^ or receiving haemodialysis [[7](#_ENREF_7)].
- *25-OH vitamin D_3_ deficiency:* as a number of methods (mass spectroscopy and immunoassay) were used over the twenty-year period covered by the study to determine 25-OH vitamin D3 deficiency, deficiency was defined by the assay used for each individual patient. The serum sample had been drawn within 1 month of treatment initiation.
- *HIV positive:* a positive result from an enzyme linked immunosorbent assay (ELISA) or nucleic acid amplification test (NAAT) for HIV type 1 infection (no cases of HIV type 2 infection were present in the final study cohort).
- *HIV negative:* either a negative ELISA / NAAT or an unknown HIV status.
- *Pulmonary cavitation:* visible on available imaging or if unavailable, recorded in the medical notes.
- *Immunosuppressant use:* receiving immunosuppressive medication (glucocorticoids such as prednisolone, oral immunosuppressants such as cyclosporine or immunosuppressive monoclonal antibody therapy such as infliximab) any time after symptom onset up until the completion of anti TB therapy.

Treatment

- *Adverse drug reaction:* any unintended adverse response to a drug occurring at a therapeutic dose and resulting in either death, drug withdrawal, change in the administration of the frequency or dose of the drug, or, that no action is required [[8](#_ENREF_8)]. These were graded by the Common Terminology Criteria for Adverse Events v4.0 [[9](#_ENREF_9)]
- *Treatment interruption:* any period in which anti-TB therapy was discontinued temporarily at the physician’s direction or due to patient action.

**Variables**

We considered a wide range of variables for inclusion in the study. Those selected for analysis were either significantly associated with recurrence in previous studies or considered biologically plausible [[5](#_ENREF_5), [10-32](#_ENREF_10)]

Demographic data obtained included age, weight, gender, housing status, ethnicity, UK birth status, tobacco and alcohol use, bacillus Calmette-Guérin vaccination status, diabetes mellitus, end stage renal failure, cancer, solid organ transplant, rheumatological conditions, HIV-1 serostatus, immunosuppressing drug use and 25-OH Vitamin D_3_ deficiency.

Laboratory values retrieved were those closest (≤ 1 month) to the date of tuberculosis diagnosis for haemoglobin, white cell count and serum creatinine. Data for pulmonary cavitation was obtained by chest-x-ray or computed tomography scanning of the thorax.

Tuberculosis-specific data obtained comprised sample acid fast staining, culture and sensitivity profile of *Mycobacterium tuberculosis* isolates; site of disease, anti-tuberculous treatment (ATT) regimen, duration of ATT, any adverse reaction to ATT and a history of ATT interruption.

**Supplementary tables**

**Table S1:Immunosuppressant medication use and indications**

| Tuberculosis  recurrence | Corticosteroid  use | Immunosuppressant  used | Stated indication for drug treatment |
| --- | --- | --- | --- |
| Yes | Yes |  | Wheeze (GP initiated prior to TB diagnosis) |
| Yes | Yes |  | ATT-induced urticaria |
| Yes | No | Methotrexate | Comorbid psoriatic arthropathy |
| Yes | Yes |  | Wheeze (GP initiated prior to TB diagnosis) |
| Yes | Yes |  | Colitis suspected before TB diagnosed; paradoxical reaction |
| Yes | Yes |  | Comorbid ulcerative colitis |
| Yes | Yes | Infliximab | Comorbid rheumatoid arthritis |
| Yes | Yes |  | Wheeze (GP initiated prior to TB diagnosis) |
| Yes | No | Methotrexate and cyclosporine | Psoriasis |
| Yes | Yes |  | Tuberculosis meningitis |
| Yes | Yes |  | Spondyloarthropathy suspected before TB diagnosed |
| Yes | Yes |  | Brittle asthma |
| Yes | Yes |  | Prevention of ureteric stricture in a renal TB case |
| Yes | Yes |  | Suspected Addison’s disease |
| Yes | Yes |  | TB IRIS |
| Yes | No | Methotrexate and cyclosporine | Rheumatoid arthritis suspected before TB diagnosed |
| Yes | Yes |  | Not stated |
| No | Yes |  | Pleural TB |
| No | Yes | Cyclophosphamide | Granulomatosis with polyangitis suspected before TB diagnosed |
| No | Yes |  | Unknown |
| No | Yes |  | Wheeze (GP initiated prior to TB diagnosis) |
| No | Yes |  | Chronic obstructive pulmonary disease |
| No | Yes |  | Disseminated TB |
| No | Yes |  | Wheeze (GP initiated prior to TB diagnosis) |
| No | Yes |  | Ocular TB |
| No | Yes |  | Suspected Addison’s disease |
| No | Yes |  | Addison’s disease |
| No | Yes | Leflunomide | Comorbid rheumatoid arthritis |
| No | Yes | Tacrolimus | Solid organ transplant |
| No | Yes |  | Addison’s disease |
| No | Yes |  | Tuberculoma |
| No | Yes |  | Tuberculoma |
| No | Yes |  | Slow to resolve pleural TB |

GP = General practitioner; ATT = Anti-tuberculous therapy; IRIS = Immune reconstitution inflammatory syndrome.

**Table S2: Site of tuberculosis disease in the primary episode in patients with and without recurrent disease**

| Site of disease | Cases (n=82) N(%) | Controls (n=164) N(%) | P value† |
| --- | --- | --- | --- |
| Pulmonary | 45 (55) | 68 (41) | - |
| Pulmonary with concomitant extrapulmonary | 10 (12) | 31 (19) | 0.09 |
| Cutaneous | 0 | 1 |  |
| Disseminated | 2 | 9 |  |
| Eye | 0 | 1 |  |
| Gastrointestinal | 1 | 1 |  |
| Lymph node | 4 | 11 |  |
| Oropharyngeal | 1 | 0 |  |
| Pleural | 2 | 6 |  |
| Spinal | 0 | 2 |  |
| Extrapulmonary | 27 (33) | 65 (40) | 0.14 |
| Bone and joint | 2 | 2 |  |
| Cutaneous | 0 | 2 |  |
| Central nervous system | 1 | 0 |  |
| Eye | 1 | 2 |  |
| Gastrointestinal | 0 | 3 |  |
| Lymph node | 17 | 37 |  |
| Peritoneal | 2 | 3 |  |
| Pleural | 2 | 6 |  |
| Psoas | 0 | 1 |  |
| Renal | 1 | 0 |  |
| Spinal | 1 | 9 |  |

†P value for the comparison of site of disease using pulmonary tuberculosis as the reference point.

**Table S3a: MIRU-VNTR genotyping data of paired isolates**

| Isolate | Type of recurrence | Lineage | Episode | ETR-A | ETR-B | ETR-C | ETR-D | ETR-E | Miru 02 | Miru 10 | Miru 16 | Miru 20 | Miru 23 | Miru 24 | Miru 26 | Miru 27 | Miru 39 | Miru 40 | Mtub04 | Mtub21 | QUB-11b | Mtub29 | Mtub30 | Mtub34 | Mtub39 | QUB-26 | QUB-4156 |
| --- | --- | --- | --- | --- | --- | --- | --- | --- | --- | --- | --- | --- | --- | --- | --- | --- | --- | --- | --- | --- | --- | --- | --- | --- | --- | --- | --- |
| 1 | Relapse | Delhi / CAS | 1st | 4 | 2 | 2 | 3 | 5 | 2 | 6 | 4 | 2 | 5 | 1 | 7 | 3 | 3 | 3 |  |  |  |  |  |  |  |  |  |
|  |  |  | 2nd | 4 | 2 | 2 | 3 | 5 | 2 | 6 | 4 | 2 | 5 | 1 | 7 | 3 | 3 | 3 |  |  |  |  |  |  |  |  |  |
| 2 | Relapse | Delhi / CAS | 1st | - | 2 | 2 | 3 | 4 | 2 | 7 | 4 | 2 | 5 | 1 | 1 | 3 | 3 | 4 |  |  |  |  |  |  |  |  |  |
|  |  |  | 2nd | - | 2 | 2 | 3 | 4 | 2 | 7 | 4 | 2 | 5 | 1 | 1 | 3 | 3 | 4 |  |  |  |  |  |  |  |  |  |
| 3 | Relapse | Delhi / CAS | 1st | - | 2 | 2 | 3 | 5 | 2 | 5 | 4 | 2 | 5 | 1 | 7 | 3 | 3 | 4 |  |  |  |  |  |  |  |  |  |
|  |  |  | 2nd | - | 2 | 2 | 3 | 5 | 2 | 5 | 4 | 2 | 5 | 1 | 7 | 3 | 3 | 4 |  |  |  |  |  |  |  |  |  |
| 4 | Relapse | Delhi / CAS | 1st | 4 | 2 | 2 | 3 | 4 | 2 | 6 | 4 | 2 | 5 | 1 | 7 | 3 | 2 | 3 |  |  |  |  |  |  |  |  |  |
|  |  |  | 2nd | 4 | 2 | 2 | 3 | 4 | 2 | 6 | 4 | 2 | 5 | 1 | 7 | 3 | 2 | 3 |  |  |  |  |  |  |  |  |  |
| 5 | Relapse | Delhi / CAS | 1st | 4 | 2 | 2 | 3 | 5 | 2 | 5 | 4 | 2 | 5 | 1 | 7 | 3 | 2 | 3 |  |  |  |  |  |  |  |  |  |
|  |  |  | 2nd | 4 | 2 | 2 | 3 | 5 | 2 | 4 | 4 | 2 | 5 | 1 | 7 | 3 | 2 | 3 |  |  |  |  |  |  |  |  |  |
| 6 | Reinfection | EAI & EAI | 1st | 5 | 6 | 2 | 2 | 6 | 2 | 6 | 3 | 2 | 6 | 2 | 2 | 3 | 1 | 3 |  |  |  |  |  |  |  |  |  |
|  |  |  | 2nd | 4 | 7 | 2 | 5 | 6 | 2 | 6 | 3 | 2 | 6 | 2 | 2 | 3 | 1 | 3 |  |  |  |  |  |  |  |  |  |
| 7 | Relapse | Delhi / CAS | 1st | 2 | 2 | 2 | 3 | 5 | 2 | 8 | 6 | 2 | 3 | 1 | 7 | 3 | 3 | 2 | 4 | 4 | 2 | 4 | 2 | 3 | 3 | 8 | 4 |
|  |  |  | 2nd | 2 | 2 | 2 | 3 | 5 | 2 | 8 | 6 | 2 | 3 | 1 | 7 | 3 | 3 | 2 | 4 | 4 | 2 | 4 | 2 | 3 | 3 | 8 | 4 |
| 8 | Relapse | Beijing | 1st | 4 | 2 | 4 | 3 | 5 | 2 | 3 | 3 | 2 | 5 | 1 | 7 | 3 | 3 | 3 |  |  |  |  |  |  |  |  |  |
|  |  |  | 2nd | 4 | 2 | 4 | 3 | 5 | 2 | 3 | 3 | 2 | 5 | 1 | 7 | - | 3 | 3 | 4 | 4 | - | 4 | 4 | 3 | 3 | 8 | 2 |
| 9 | Reinfection | Delhi / CAS | 1st | 4 | 2 | 2 | 3 | 5 | 2 | 4 | 5 | 2 | 5 | 1 | 7 | 3 | 3 | 4 | 4 | 4 | 2 | 4 | 2 | 3 | 3 | 1 | 4 |
|  |  |  | 2nd | 4 | 2 | 2 | 3 | 5 | 2 | 4 | 5 | 2 | 5 | 1 | 7 | 3 | 3 | - | 4 | 4 | 2 | 4 | 2 | 3 | 3 | 7 | 4 |
| 10 | Relapse | Delhi / CAS | 1st | 4 | 2 | 2 | 3 | 5 | 2 | 8 | 4 | 2 | 5 | 1 | 7 | 3 | 3 | 2 | 3 | 4 | 2 | 4 | 2 | 3 | 3 | 7 | 4 |
|  |  |  | 2nd | 4 | 2 | 2 | 3 | 5 | 2 | - | 4 | 2 | 5 | 1 | 7 | 3 | 3 | 2 | 3 | 4 | 2 | 4 | 2 | 3 | 3 | 7 | 4 |

From 2003-2010, isolates underwent 15 locus MIRU-VNTR and 24 locus MIRU-VNTR from 2010 onwards. Isolates were assigned lineages using the MIRU-VNTRplus database[[33](#_ENREF_33)] (http://www.miru-vntrplus.org). MIRU-VNTR= Mycobacterial Interspersed Repetitive Units- Variable Number Tandem Repeat typing; CAS=Central Asian Strain; EAI=East African Indian (TB strain-type).

| Isolate | Type of recurrence | Lineage | Episode | ETR-A | ETR-B | ETR-C | ETR-D | ETR-E | Miru 02 | Miru 10 | Miru 16 | Miru 20 | Miru 23 | Miru 24 | Miru 26 | Miru 27 | Miru 39 | Miru 40 | Mtub04 | Mtub21 | QUB-11b | Mtub29 | Mtub30 | Mtub34 | Mtub39 | QUB-26 | QUB-4156 |
| --- | --- | --- | --- | --- | --- | --- | --- | --- | --- | --- | --- | --- | --- | --- | --- | --- | --- | --- | --- | --- | --- | --- | --- | --- | --- | --- | --- |
| 11 | Relapse | Delhi / CAS | 1st | 4 | 2 | 2 | 3 | 5 | 2 | 6 | 4 | 2 | 5 | 1 | 3 | 3 | 3 | 3 |  |  |  |  |  |  |  |  |  |
|  |  |  | 2nd | 4 | 2 | 2 | 3 | 5 | 2 | 6 | 4 | 2 | 5 | 1 | 3 | 3 | 3 | 3 |  |  |  |  |  |  |  |  |  |
| 12 | Relapse | EAI | 1st | - | 4 | 2 | 6 | 5 | 2 | 4 | 3 | 2 | 6 | 2 | 2 | 3 | 1 | 3 |  |  |  |  |  |  |  |  |  |
|  |  |  | 2nd | - | 4 | 2 | - | 5 | 2 | 4 | 3 | 2 | 6 | 2 | 2 | 3 | 1 | 3 | 2 | - | 3 | 3 | 2 | 3 | 7 | 4 | 1 |
| 13 | Relapse | Delhi / CAS | 1st | - | 2 | 2 | 3 | 4 | 2 | 7 | 4 | 2 | 5 | 1 | 1 | 3 | 3 | 4 |  |  |  |  |  |  |  |  |  |
|  |  |  | 2nd | - | 2 | 2 | 3 | 4 | 2 | 7 | 4 | 2 | 5 | 1 | 1 | 3 | 3 | 4 | 4 | 3 | 2 | 4 | 2 | 2 | 2 | 5 | 4 |
| 14 | Relapse | Delhi / CAS | 1st | 3 | 2 | 2 | 3 | 4 | 2 | 7 | 4 | 2 | 5 | 1 | 1 | 3 | 3 | 4 |  |  |  |  |  |  |  |  |  |
|  |  |  | 2nd | - | 2 | 2 | 3 | 4 | 2 | 7 | 4 | 2 | 5 | 1 | 1 | 3 | 3 | 4 |  |  |  |  |  |  |  |  |  |
| 15 | Relapse | Delhi / CAS | 1st | 4 | 2 | 2 | 3 | 4 | 2 | 7 | 4 | 2 | 5 | 1 | 1 | 3 | 3 | 4 |  |  |  |  |  |  |  |  |  |
|  |  |  | 2nd | - | 2 | 2 | 3 | 4 | 2 | 7 | 4 | 2 | 5 | 1 | 1 | 3 | 3 | 4 |  |  |  |  |  |  |  |  |  |
| 16 | Relapse | EAI | 1st | 4 | 2 | 4 | 5 | 4 | 2 | 5 | 3 | 2 | 6 | 2 | 2 | 3 | 3 | 5 |  |  |  |  |  |  |  |  |  |
|  |  |  | 2nd | 4 | 2 | 4 | 5 | 4 | 2 | 5 | 3 | 2 | 6 | 2 | 2 | 3 | 3 | 5 |  |  |  |  |  |  |  |  |  |
| 17 | Reinfection | EAI & EAI | 1st | 6 | 1 | 4 | 6 | 5 | 2 | 4 | 2 | 2 | 6 | 2 | 2 | 3 | 3 | 4 |  |  |  |  |  |  |  |  |  |
|  |  |  | 2nd | 7 | 1 | 4 | 4 | 4 | 2 | 4 | 2 | 2 | 5 | 2 | 2 | 3 | 3 | 4 |  |  |  |  |  |  |  |  |  |
| 18 | Relapse | Delhi / CAS | 1st | - | 2 | 2 | 3 | 4 | 2 | 7 | 5 | 2 | 5 | 1 | 7 | 4 | 3 | 3 |  |  |  |  |  |  |  |  |  |
|  |  |  | 2nd | - | 2 | 2 | 3 | 4 | 2 | 7 | 5 | 2 | 5 | 1 | 7 | 4 | 3 | 3 | 4 | 4 | - | 4 | 4 | 3 | 3 | 8 | 2 |
| 19 | Relapse | EAI | 1st | 4 | 4 | 2 | 6 | 5 | 2 | 4 | 3 | 2 | 6 | 2 | 2 | 3 | 1 | 3 |  |  |  |  |  |  |  |  |  |
|  |  |  | 2nd | 4 | 4 | 2 | 6 | 5 | 2 | 4 | 3 | 2 | 6 | 2 | 2 | 3 | 1 | 3 |  |  |  |  |  |  |  |  |  |

**Table S3b: Remaining MIRU-VNTR genotyping data of paired isolates**

From 2003-2010, isolates underwent 15 locus MIRU-VNTR and 24 locus MIRU-VNTR from 2010 onwards. Isolates were assigned lineages using the MIRU-VNTRplus database[[33](#_ENREF_33)] (http://www.miru-vntrplus.org). MIRU-VNTR= Mycobacterial Interspersed Repetitive Units- Variable Number Tandem Repeat typing. CAS=Central Asian Strain; EAI=East African Indian (TB strain-type).

**Table S4: Demographics of cases of relapse and reinfection**

|  | **Relapse (n=16) N (%)** | **Reinfection (n=3) N(%)** | **P value** |
| --- | --- | --- | --- |
| Age (year, +/-SD) | 39.8 (+/-18.4) | 31.3 (+/-3.2) | 0.58 |
| Weight (kg) | 48.0 (40.5-67.5)** | 50.0 (44.0-57.0)** | 0.91 |
| Gender |  |  |  |
| Male | 8 (50.0) | 1 (33.3) | - |
| Female | 8 (50.0) | 2 (66.6) | 1.00 |
| Ethnicity |  |  |  |
| White | 2 (12.5) | 0 | - |
| Afro-Caribbean | 1 (6.3) | 0 | - |
| Indian subcontinent | 11 (68.7) | 3 (100) | 1.00 |
| Other | 2 (12.5) | 0 | - |
| Housed | 16 (100) | 3 (100) | 1.00 |
| UK Born^a^ | 7 (43.8) | 0 | 0.50 |
| Habitual alcohol consumption^b^ | 3 (20) | 0 | 1.00 |
| Habitual tobacco smoking^c^ | 5 (33.0) | 0 | 0.52 |
| BCG vaccinated^d^ | 6 (85.7) | 1 (100) | 1.00 |
| Comorbidities |  |  |  |
| Diabetes mellitus | 3 (1.8) | 0 | 1.00 |
| Rheumatological conditions | 1 (6.3) | 0 | - |
| Vitamin D deficiency^e^ | 4 (30.1) | 0 | 1.00 |
| Laboratory |  |  |  |
| Haemoglobin (g/dl +/-SD) | 11.2 (+/-1.8) | 11.16 (+/-3.3) | 0.96 |
| White cell count (x10^9^ cells/L) | 9.2 (6.6-10.4)** | 7.8 (7.2-12.)** | 0.74 |
| Creatinine (µmol/l) | 71.0 (62.0-81.0) ** | 61.0 (54.0-67.0)** | 0.16 |
| Primary disease site |  |  |  |
| PTB | 12 (75.0) | 1 (33.3) |  |
| PTB + EPTB | 1 (6.3) | 0 | 1.00 |
| EPTB | 3 (18.7) | 2 (66.6) | 0.17 |
| Pulmonary cavitation | 8 (50) | 0 | 0.23 |
| Microbiology |  |  |  |
| AFB Smear positive | 9 (56.3) | 0 | 0.21 |
| Drug resistance | 4 (25.0) | 1 (33.3) | 1.00 |
| Treatment |  |  |  |
| Standard regimen | 9 (56.3) | 2 (66.6) | 1.00 |
| Adverse drug reaction | 6 (37.5) | 0 | 0.52 |
| No reaction | 10(62.5) | 3(100) |  |
| Grade 1 / 2 | 1(6.3) | 0 | 0.79 |
| Grade 3 / 4 | 5 | 0 | 0.52 |
| Treatment interruption (days) | 5.79(+/-15.8) | 0 | 0.43 |
| Immunosuppressing drugs | 3 (18.7) | 0 | 1.00 |
| Duration (days) | 259 (168-323)** | 168 (168-366)** | 0.56 |

Missing data relapse and reinfection: a: 0&1; b:1&0; c: 1&0; d:9&3; e:3&2.

SD= standard deviation; BCG= Bacillus Calmette-Guérin; CXR=chest x-ray; AFB= acid fast bacillus. **median and IQR

**References**

1. Definitions and reporting framework for tuberculosis – 2013 revision. In*.*: World Health Organization; 2014.

2. Lambert ML, Hasker E, Van Deun A, Roberfroid D, Boelaert M, Van der Stuyft P: Recurrence in tuberculosis: relapse or reinfection? *The Lancet infectious diseases* 2003, 3(5):282-287.

3. Narayanan S, Swaminathan S, Supply P, Shanmugam S, Narendran G, Hari L, Ramachandran R, Locht C, Jawahar MS, Narayanan PR: Impact of HIV infection on the recurrence of tuberculosis in South India. *The Journal of infectious diseases* 2010, 201(5):691-703.

4. Office for National Statistics. Ethnic Group statistics: A Guide for the Collection and Classification of Ethnicity Data. In*.* London: ONS; 2009.

5. Lee PH, Lin HC, Huang AS, Wei SH, Lai MS, Lin HH: Diabetes and risk of tuberculosis relapse: nationwide nested case-control study. *PloS one* 2014, 9(3):e92623.

6. (2) Classification and diagnosis of diabetes. *Diabetes care* 2015, 38 Suppl:S8-S16.

7. K/DOQI clinical practice guidelines for chronic kidney disease: evaluation, classification, and stratification. *American journal of kidney diseases : the official journal of the National Kidney Foundation* 2002, 39(2 Suppl 1):S1-266.

8. Van der Walt M, Lancaster J, Odendaal R, Davis JG, Shean K, Farley J: Serious treatment related adverse drug reactions amongst anti-retroviral naive MDR-TB patients. *PloS one* 2013, 8(4):e58817.

9. Common Terminology Criteria for Adverse Events v4.0 (CTCAE). In*.*; 2009.

10. Goswami A, Chakraborty U, Mahapatra T, Mahapatra S, Mukherjee T, Das S, Das A, Dey SK, Ray S, Bhattacharya B *et al*: Correlates of Treatment Outcomes and Drug Resistance among Pulmonary Tuberculosis Patients Attending Tertiary Care Hospitals of Kolkata, India. *PloS one* 2014, 9(10):e109563.

11. Crofts JP, Andrews NJ, Barker RD, Delpech V, Abubakar I: Risk factors for recurrent tuberculosis in England and Wales, 1998-2005. *Thorax* 2010, 65(4):310-314.

12. Selassie AW, Pozsik C, Wilson D, Ferguson PL: Why pulmonary tuberculosis recurs: a population-based epidemiological study. *Annals of epidemiology* 2005, 15(7):519-525.

13. Anaam MS, Ibrahim MI, Al Serouri AW, Bassili A, Aldobhani A: A nested case-control study on relapse predictors among tuberculosis patients treated in Yemen's NTCP. *Public health action* 2012, 2(4):168-173.

14. Sahebi L, Ansarin K, Maryam S, Monfaredan A, Sabbgh Jadid H: The factors associated with tuberculosis recurrence in the northwest and west of iran. *The Malaysian journal of medical sciences : MJMS* 2014, 21(6):27-35.

15. Leung CC, Chan CK, Chang KC, Law WS, Lee SN, Tai LB, Leung EC, Tam CM: Immigrants and tuberculosis in Hong Kong. *Hong Kong medical journal = Xianggang yi xue za zhi / Hong Kong Academy of Medicine* 2015, 21(4):318-326.

16. Millet JP, Orcau A, de Olalla PG, Casals M, Rius C, Cayla JA: Tuberculosis recurrence and its associated risk factors among successfully treated patients. *Journal of epidemiology and community health* 2009, 63(10):799-804.

17. Brudney K, Dobkin J: Resurgent tuberculosis in New York City. Human immunodeficiency virus, homelessness, and the decline of tuberculosis control programs. *The American review of respiratory disease* 1991, 144(4):745-749.

18. Li F, Song CY, Zhao F, Liang ML, Liu ZM, Guo XY, Wang Y, He GX: Follow-up Study of Retreatment TB Patients with Sputum Smear and/or Culture Positive Two Years after They were Declared Cured with First-line Anti-TB Drugs in Shandong Province. *Biomedical and environmental sciences : BES* 2015, 28(2):152-156.

19. Thomas A, Gopi PG, Santha T, Chandrasekaran V, Subramani R, Selvakumar N, Eusuff SI, Sadacharam K, Narayanan PR: Predictors of relapse among pulmonary tuberculosis patients treated in a DOTS programme in South India. *The international journal of tuberculosis and lung disease : the official journal of the International Union against Tuberculosis and Lung Disease* 2005, 9(5):556-561.

20. Leung CC, Yew WW, Chan CK, Chang KC, Law WS, Lee SN, Tai LB, Leung EC, Au RK, Huang SS *et al*: Smoking adversely affects treatment response, outcome and relapse in tuberculosis. *The European respiratory journal* 2014.

21. Yen YF, Yen MY, Lin YS, Lin YP, Shih HC, Li LH, Chou P, Deng CY: Smoking increases risk of recurrence after successful anti-tuberculosis treatment: a population-based study. *The international journal of tuberculosis and lung disease : the official journal of the International Union against Tuberculosis and Lung Disease* 2014, 18(4):492-498.

22. Sonnenberg P, Murray J, Glynn JR, Shearer S, Kambashi B, Godfrey-Faussett P: HIV-1 and recurrence, relapse, and reinfection of tuberculosis after cure: a cohort study in South African mineworkers. *Lancet* 2001, 358(9294):1687-1693.

23. Pascopella L, Deriemer K, Watt JP, Flood JM: When tuberculosis comes back: who develops recurrent tuberculosis in california? *PloS one* 2011, 6(11):e26541.

24. Chang KC, Leung CC, Yew WW, Ho SC, Tam CM: A nested case-control study on treatment-related risk factors for early relapse of tuberculosis. *American journal of respiratory and critical care medicine* 2004, 170(10):1124-1130.

25. Hung CL, Chien JY, Ou CY: Associated factors for tuberculosis recurrence in Taiwan: a nationwide nested case-control study from 1998 to 2010. *PloS one* 2015, 10(5):e0124822.

26. Mehta S, Mugusi FM, Bosch RJ, Aboud S, Urassa W, Villamor E, Fawzi WW: Vitamin D status and TB treatment outcomes in adult patients in Tanzania: a cohort study. *BMJ open* 2013, 3(11):e003703.

27. Quy HT, Lan NT, Borgdorff MW, Grosset J, Linh PD, Tung LB, van Soolingen D, Raviglione M, Co NV, Broekmans J: Drug resistance among failure and relapse cases of tuberculosis: is the standard re-treatment regimen adequate? *The international journal of tuberculosis and lung disease : the official journal of the International Union against Tuberculosis and Lung Disease* 2003, 7(7):631-636.

28. Guerra-Assuncao JA, Houben RM, Crampin AC, Mzembe T, Mallard K, Coll F, Khan P, Banda L, Chiwaya A, Pereira RP *et al*: Recurrence due to Relapse or Reinfection With Mycobacterium tuberculosis: A Whole-Genome Sequencing Approach in a Large, Population-Based Cohort With a High HIV Infection Prevalence and Active Follow-up. *The Journal of infectious diseases* 2015, 211(7):1154-1163.

29. El Sahly HM, Wright JA, Soini H, Bui TT, Williams-Bouyer N, Escalante P, Musser JM, Graviss EA: Recurrent tuberculosis in Houston, Texas: a population-based study. *The international journal of tuberculosis and lung disease : the official journal of the International Union against Tuberculosis and Lung Disease* 2004, 8(3):333-340.

30. Hamilton CD, Stout JE, Goodman PC, Mosher A, Menzies R, Schluger NW, Khan A, Johnson JL, Vernon AN: The value of end-of-treatment chest radiograph in predicting pulmonary tuberculosis relapse. *The international journal of tuberculosis and lung disease : the official journal of the International Union against Tuberculosis and Lung Disease* 2008, 12(9):1059-1064.

31. Fitzgerald DW, Desvarieux M, Severe P, Joseph P, Johnson WD, Jr., Pape JW: Effect of post-treatment isoniazid on prevention of recurrent tuberculosis in HIV-1-infected individuals: a randomised trial. *Lancet* 2000, 356(9240):1470-1474.

32. Nahid P, Gonzalez LC, Rudoy I, de Jong BC, Unger A, Kawamura LM, Osmond DH, Hopewell PC, Daley CL: Treatment outcomes of patients with HIV and tuberculosis. *American journal of respiratory and critical care medicine* 2007, 175(11):1199-1206.

33. Allix-Beguec C, Harmsen D, Weniger T, Supply P, Niemann S: Evaluation and strategy for use of MIRU-VNTRplus, a multifunctional database for online analysis of genotyping data and phylogenetic identification of Mycobacterium tuberculosis complex isolates. *Journal of clinical microbiology* 2008, 46(8):2692-2699.
